# Degradation Susceptibility of Al-2.18Mg-1.92Li Alloy in Severe Environmental Conditions

**DOI:** 10.3390/ma18091938

**Published:** 2025-04-24

**Authors:** Franjo Kozina, Zdenka Zovko Brodarac, Mitja Petrič, Barbara Šetina Batič

**Affiliations:** 1 Faculty of Metallurgy, University of Zagreb, 44 000 Sisak, Croatia; 2 Faculty of Natural Sciences and Engineering, University of Ljubljana, 1000 Ljubljana, Slovenia; mitja.petric@ntf.uni-lj.si; 3 Institute of Metals and Technology, 1000 Ljubljana, Slovenia; barbara.setina@imt.si

**Keywords:** aluminum–magnesium–lithium alloys, microstructure development, severe environmental conditions, degradation, dealloying

## Abstract

Due to the specific application of aluminum–magnesium–lithium (Al-Mg-Li) alloys in the transportation industry, it is necessary to consider the influence of microstructure development on material degradation under severe environmental conditions. This degradation was simulated according to the standard test method ASTM G34-01 (2018) on a newly designed and synthesized Al-2.1Mg-1.92Li alloy in the as-cast condition. The degradation susceptibility of the alloy was estimated by measuring the changes in the sample mass and microhardness, and the pH and chemical composition of the environment with respect to the exposure time. The influence of the microstructure constituents on the degradation of the alloy was determined using metallographic analysis of the exposed surface and cross-section of the samples after testing. During the degradation, dealloying of the α_Al_ matrix through Li, Mg and Al component dissolution resulted in a decrease in the mass of the samples, an increase in the pH of the environment and changes in its chemical composition. This observation was also confirmed by the results of the metallographic analysis. The degradation involved the formation of cavities around the Al_8_Mg_5_ (β) and Al_2_LiMg (T) intermetallic phases through an anodic dissolution mechanism. The increase in microhardness values after exposure indicated an increase in the stress around the degradation front due to the wedge effect of the degradation products. The results of the investigation support the potential application of the synthesized Al-2.1Mg-1.92Li alloy under the severe environmental conditions defined by the ASTM G34-01 (2018) standard.

## 1. Introduction

Since the middle of the 20th century, the transport sector has played a crucial role in globalization by connecting people, companies, goods and markets. This increased mobility has led to a growing demand for vehicles that are safer, faster, more efficient, more cost-effective and more environmentally friendly [1,2,3]. The necessary performance requirements can be met by implementing ecodesign principles in vehicle production [4] and developing alloys with improved functional properties [5]. Ecodesign is a comprehensive approach to product design and development that focuses on minimizing environmental impacts throughout a product’s life cycle. This approach encourages manufacturers and companies to proactively comply with regulations and remain competitive [6]. From a product design standpoint, material selection and development require the precise definition of key guidelines, such as cost versus lightweight, weight versus durability and functionality versus recyclability [7]. The modification of materials’ structural composition and manufacturing technology requires the consideration of performance, environmental and economic factors [8]. As an innovative material, aluminum alloys (AAs) were first introduced to the automotive industry in the 1980s with the launch of the aluminum-framed Audi A8. A noticeable increase in vehicle performance, and improvement in fuel economy and gas emissions cumulated in the production of the Ford F-150 with an all-aluminum body [9]. In addition to traditional fuel-powered vehicles, weight reduction is the key parameter in the development of modern electric means of transportation [10]. Since the first flight of the Wright Flyer in 1903, in which an aluminum alloy with 8.0 wt.% copper (Cu) was used for the crankcase of the engine [11], AAs have played a central role in the aviation industry. Later, in 1969, an AA with 6.3 wt.% Cu was used to manufacture the shuttle boosters that enabled humankind’s first walk on the moon [9]. Today, the aerospace industry is focused on developing lightweight components with higher strength, better performance at elevated temperatures and improved corrosion resistance [12]. These advances are helping to reduce the number of components and lower vehicle manufacturing costs [13].

In the transportation industry, both cast and wrought AAs are used for vehicle production. Cast AAs with silicon (Si) as the primary alloying element (3xx.x) are used in the automotive industry for the fabrication of products with high safety requirements [2,3,14]. Wrought AAs from the 2xxx and 7xxx series are used in the manufacture of structural components for the aerospace industry [15]. In the 2xxx series, Cu is the primary alloying addition, which is often combined with magnesium (Mg) to increase toughness and damage tolerance [16,17,18,19,20]. In the 7xxx series, the high strength and stiffness are achieved by alloying with Mg, zinc (Zn) and Cu [21]. Although the mechanical and functional properties of the 3xx.x, 2xxx and 7xxx alloy systems are the result of microstructure development during solidification [2,3,17,18,19,22,23] or subsequent processing [24,25], the formation and coarsening of complex intermetallic phases can affect their applicability [2,3,26]. The addition of lithium (Li) can improve the elastic modulus, specific strength, fracture toughness and fatigue crack growth resistance and further reduce the weight [27]. By redesigning the chemical composition through the addition of Li, in conjunction with the thermodynamic and processing parameters [28], the solidification sequence is directly influenced, determining the type, morphology, amount and distribution of the intermetallic phases [29].

The development of the microstructure in aluminum–lithium (Al-Li) alloys results from the decreasing solubility of Li in the α_Al_ solid solution during solidification. The maximum solubility of Li in the α_Al_ solid solution is 4.0 wt.% at a temperature of 603.0 °C when the eutectic reaction occurs. At a temperature of 100.0 °C, the solid solubility of Li decreases to less than 1.0 wt.% and allows for the solidification of the intermetallic phases [30]. In this alloying system, the metastable Al_3_Li (δ’) phase is the primary strengthening precipitate. Compared to the intermetallic phases Al_2_CuMg (S), AlxCu_4_Mg_5_Si_4_ (W) and Al_32_(Mg,Zn)_49_ (T), which are characteristic of the wrought 2xxx and 7xxx AA series, the Al_3_Li (δ’) phase maintains a favorable morphology at higher processing temperatures and longer soaking times [31]. The preferential precipitation of the Al_3_Li (δ’) phase can be induced by adding Mg. The addition of Mg increases the amount of the Al_3_Li (δ’) phase by lowering the solubility of Li in the α_Al_ solid solution and partly replacing Li atoms in the phase lattice of Al_3_Li (δ’). The solidification and microstructure development in Al-Mg-Li alloys vary depending on the Li/Mg ratio and include the formation of Al_8_Mg_5_ (β), Al_12_Mg_17_ (γ), Al_2_LiMg (T), AlLi (δ) and Al_3_Li (δ’) phases, as described in Equations (1) and (2) [32].

High Li/Mg ratio α_Al_ matrix → Al_3_Li (δ’) → AlLi (δ), (1)

Low Li/Mg ratio α_Al_ matrix → Al_3_Li (δ’) → Al_2_LiMg (T). (2)

**Figure 1:**
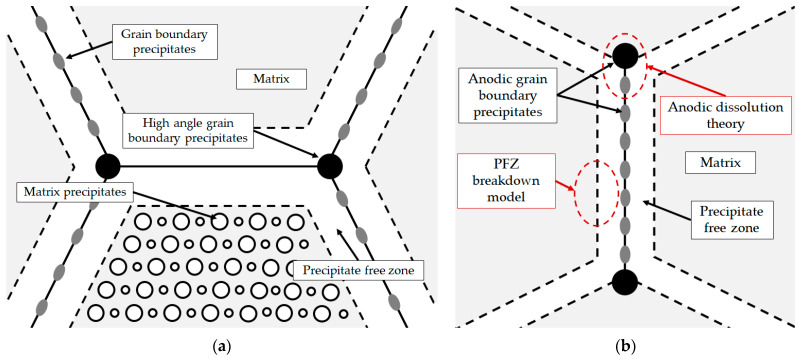
Schematic of (**a**) Al-Li-Mg alloy microstructure development [35] and (**b**) microstructure degradation process [36].

At a high Li/Mg ratio (Equation (1)), the microstructure development begins with the eutectic solidification of the α_Al_ matrix and the Al_3_Li (δ’) phase. The solidification of the stable AlLi (δ) phase at the α_Al_ grain boundaries occurs during the peritectic reaction with the participation of the previously solidified metastable Al_3_Li (δ’) phase and the bulk liquid (L) [33]. On the other hand, the peritectic reaction in the AA with a low Li/Mg ratio (Equation (2)), leads to solidification of the ternary Al_2_LiMg (T) phase at the high-angle grain boundaries. In both cases, the involvement of the Al_3_Li (δ’) phase in the peritectic reaction and the development of other microstructure constituents leads to the formation of precipitate-free zones (PFZs) near the α_Al_ grain boundaries (Figure 1a). The amount of grain boundary precipitates is also increased by the Al_8_Mg_5_ (β) and Al_12_Mg_17_ (γ) phase solidification. The peritectic reaction involving the Al_2_LiMg (T) phase and the Mg bulked liquid (L) results in the solidification of the Al_12_Mg_17_ (γ) phase, which hinders the α_Al_ grain boundary mobility and reduces the grain size [32]. The microstructure development ends with the solidification of the brittle and irregular Al_8_Mg_5_ (β) phases and the Mg bulked α_Al_ matrix due to bulking of the liquid phase (L) with Mg (Figure 1a) [34].

The high amount of grain boundary precipitates and the formation of PFZs increase the plasticity and the stress around the grain boundaries, negatively affecting the mechanical properties and the degradation stability [37]. Depending on the chemical composition of the environment, Al-Li-Mg alloys are highly inclined to cavity formation, intergranular degradation and exfoliation. Cavities form when Al-Li-Mg alloys are exposed to environments with a pH value outside the range of 3 to 10. The cavities can serve as an initiator for intergranular and exfoliation degradation [38]. The intergranular degradation process primarily targets the grain boundaries and the surrounding α_Al_ matrix and can be explained by the anodic dissolution theory and the PFZ breakdown model (Figure 1b) [39]. While the anodic dissolution mechanism involves the interaction between the anodic Al_8_Mg_5_ (β), Al_12_Mg_17_ (γ), Al_2_LiMg (T), AlLi (δ) and Al_3_Li (δ’) intermetallic phases and the α_Al_ matrix, the PFZ breakdown model is based on the difference in electrochemical potential between PFZs and the bulked α_Al_ matrix (Figure 1b) [40]. Exfoliation degradation occurs in thermomechanically processed alloys with a highly directional grain structure. During exfoliation degradation, the Al from the α_Al_ matrix is converted into the insoluble, very voluminous hydrate aluminum oxide. Further attacks and the progression of degradation are made possible by the wedge effect of the degradation products [41].

This study analyzed the degradation mechanism of a synthesized Al-2.18Mg-1.92Li alloy and its microstructure behavior under the severe degradation conditions described in ASTM G34-01 “Standard Test Method for Exfoliation Corrosion Susceptibility of 2xxx and 7xxx Series of Aluminum Alloys (EXCO test)” [42]. This method requires the continuous immersion of the samples in a solution with a defined chemical composition (EXCO solution) that simulates different types of outdoor exposure conditions, especially in marine, industrial and transportation environments. Degradation stability of the alloy was determined by measuring the sample mass and the chemical composition of the solution after 5, 24, 48 and 72 h of degradation. The effect of microstructure development on the accelerated degradation process was determined using various metallographic analysis techniques. The degradation mechanism was defined by comparing the initial microstructure with the microstructure of the samples after degradation. The impact of both the dealloying and wedge effect on the mechanical properties of the degradation affected zone was estimated using microhardness measurements.

## 2. Materials and Methods

**Figure 2:**
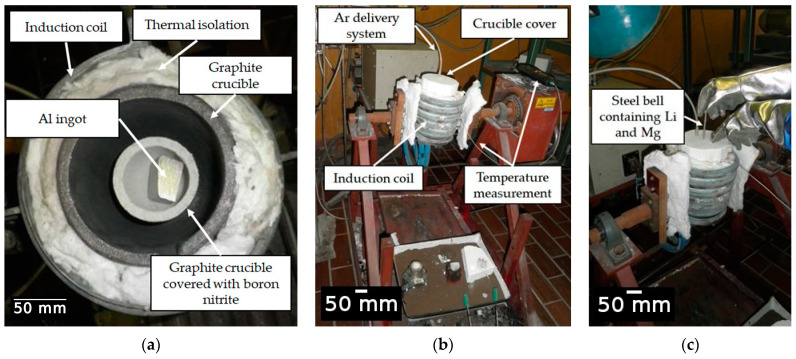
The experimental setup for alloy synthesis: (**a**) positioning of graphite crucible and raw material, (**b**) preparation for synthesis and (**c**) addition of alloying elements via a steel bell.

The Al-2.18Mg-1.92Li alloy was synthesized in an induction melting furnace using a protective argon (Ar) atmosphere and a complete crucible cover (Figure 2). The technically pure Al ingot (99.8%) was mechanically cleaned using a wire brush and placed in a graphite crucible. The protective atmosphere was generated using compressed Ar with the designation UN 1006 and a pressure of 10.0 MPa. Before they were placed into a steel bell, a Mg rod (99.98% purity) and a Li rod (99.8% purity) were wrapped in commercially pure Al foil (Figure 2b). The graphite crucible and the steel bell were coated in born nitrite to prevent crucible attack and contamination of the melt. The aluminum ingot melted at a temperature of about 690.0 °C. The Mg and Li were added at 720.0 °C by immersing the steel bell (Figure 2c). To offset the reduction in temperature resulting from the addition of alloying elements, the melt was heated to 720.0 °C. A steel rod was used to collect the small number of oxides that had floated to the surface of the melt. The sample was then cast in the open atmosphere and solidified in a permanent steel mold.

The chemical composition of the synthesized alloy was determined spectroscopically using an inductively coupled plasma mass spectrometer ICP-MS, Agilent Technologies, Santa Clara, CA, USA. The sample for the ICP-MS analysis was obtained by dissolving the sample shavings in the standard solution. Prior to the measurements, the spectrometer was calibrated using reference solutions of 1.0, 10.0, 50.0 and 100.0 mg/m^3^ Li to ensure an accuracy of 97.0% for the measurements.

The thermodynamic calculations under equilibrium and non-equilibrium conditions and the reactions in the liquid and solid states were calculated using Thermo-Calc 2022a software Thermo-Calc Software AB, Solna, Sweeden. The solidification sequence was calculated with the “TCAL68: Al Alloys v8.1” technical sheet for Al. Calculations under both equilibrium and non-equilibrium conditions were performed with respect to the weight percentages of Al, Mg and Li in the 20.0 to 780.0 °C temperature interval at a pressure of 1 10^5^ Pa for 1 g of the melt. The equilibrium calculations were used to construct the equilibrium phase diagram and to predict all the thermodynamically stable phases as a function of temperature. The stability of the phases was estimated by calculating the changes in Gibbs energy as a function of temperature. The one axis equilibrium calculations were used to evaluate the component (Al, Mg and Li) distribution in all the phases and to determine the dependence of the Gibbs energy on the mass fraction of the components at a temperature of 25.0 °C. The thermodynamic calculations under non-equilibrium conditions were calculated based on the classical Scheil–Gulliver model. This thermodynamic model assumes that diffusion in the liquid phase is infinitely fast, that there is no diffusion in the solid phase and that the interface between the liquid and solid phases is in thermodynamic equilibrium. The Scheil calculator was used to estimate the solidification range of the alloy and the composition of the final solidifying liquid. In addition to determining the solidification sequence and microstructure constituent development under equilibrium and non-equilibrium conditions, the results of the Thermo Calc 2022a calculations were used to estimate the possible degradation mechanism of the synthesized Al-2.18Mg-1.92Li alloy. Based on the Gibbs energy and the data available in the literature, the stability of the intermetallic phases compared to the matrix was analyzed. The results of the component redistribution between all the phases were used to estimate the possibility of PFZ formation and the occurrence of matrix bulking with Mg and Li during solidification. The correlation of the Gibbs energy with the mass fraction of the components at room temperature, at which the EXCO test was performed, allowed for the evaluation of microstructural degradation due to dealloying.

The accelerated degradation process based on chemical and electrochemical reactions was used to simulate the degradation susceptibility of the Al-2.18Mg-1.92Li alloy by exposing the samples to an EXCO solution. The solution was prepared by dissolving 324.0 g of sodium chloride (NaCl) and 50.0 g of potassium nitrite (KNO_3_) in water. The solution was diluted to 1.0 l with distilled water after 6.3 mL of HNO_3_ had been added. The resulting solution had an initial pH of 0.4 ± 0.1. To avoid the impact of degradation products on the overall solution chemistry, around 200.0 mL of solution was used per sample.

**Figure 3:**
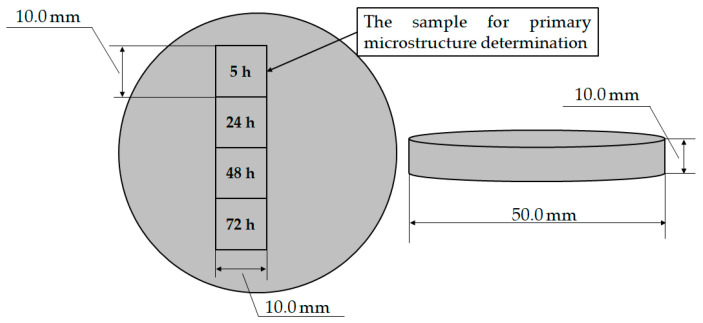
Samples for EXCO testing.

The four samples for degradation testing were obtained by cutting the sample with a circular geometry and diameter of Ø 50.0 mm and thickness of 10.0 mm (Figure 3).

**Figure 4:**
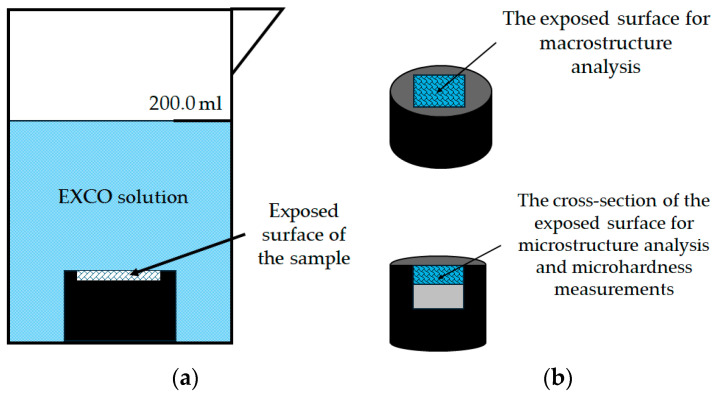
The experimental setup: (**a**) sample during exposure to severe environment and (**b**) illustration of the surface analysis after exposure.

Before exposure, the samples were mounted in a conductive material at 180.0 °C and 250.0 bar using mounting equipment CitoPress-30, Struers, Copenhagen, Denmark. Mounting was performed to limit the degradation effect of the EXCO solution to only one exposed surface (Figure 4a). The mounted samples were automatically prepared using standard metallographic preparation techniques with grinding and polishing machine Tegramin-30, Struers, Copenhagen, Denmark. The parameters used to evaluate the degradation susceptibility of the samples under severe degradation conditions are listed in Table 1.

After mounting and standard metallographic preparation, the samples were weighed to determine the initial mass of the sample (m_S_) using a laboratory analytical balance Mettler Toledo, Columbus, OH, USA with a measurement error of ±1.0 10^−3^ g. The metallographic analysis was performed on one of the samples (Figure 3, exposure time of 5 h) to determine the microstructure before degradation. The mass of the degraded samples (*m_E_*) was measured after the samples were neutralized in concentrated nitric acid (HNO_3_), rinsed with water and alcohol and dried in hot air (Table 1). Equation (3) was used to calculate the degradation rate of the samples:

(3) $$v_{cor}=\frac{∆m}{t_{exp}},$$

where ∆m is the difference between the initial and final mass of the samples and $t_{exp}$ is the exposure time (Table 1). The influence of sample dealloying on the degradation environment was assessed by determening the pH and chemical composition of the EXCO solution (Table 1). The initial pH (pH_S_) and final pH (pH_E_) of the EXCO solution were measured using a laboratory pH meter and conductometer infoLab LeV1, Mettler Toledo, Columbus, OH, USA. The increase in the Al, Mg and Li contents in the EXCO solution after degradation of the samples was determined spectroscopically using ICP-MS. The chemical composition was analyzed using an spectrometer Agilent Technologies, Santa Clara, CA, USA). Each sample of EXCO solution was diluted with standardized solutions to minimize the effects of high sodium (Na) and chlorine (Cl) concentrations and to ensure 97.0% accuracy. This made it possible to correlate the degradation rate with the changes in the properties of the EXCO solution.

The metallographic analysis was performed in order to identify the microstructural constituents in the samples before degradation and to estimate the impact of the severe environmental conditions on microstructure of the samples. The macrostructure of the sample was observed after etching using Barker’s anodizing method to reveal the grain structure. The etching solution was prepared by mixing 5.0 mL of fluoroboric acid (HBF_4_) in 200.0 mL of distilled water. Etching was performed by applying a current with an intensity of 20.0 V to the sample–platinum counter electrode system over a period of 120 s. To identify the intermetallic phases, the initial microstructure of the sample was analyzed in the polished and etched condition. The samples were color-etched with the Wecks etching solution, which was prepared by dissolving 10.0 g of potassium permanganate (KMnO_4_) and 1.0 g of sodium hydroxide (NaOH) in 250.0 mL of distilled water. The samples were etched for approximately 5 s.

Following exposure, the macrostructure and microstructure of the exposed surface were analyzed without prior metallographic preparation (Figure 4b). The samples were then cut and their cross-sectional surfaces were prepared using standard metallographic techniques, including grinding and polishing. The cross-section surface was examined in the polished condition (Figure 4b). Light microscopy of the exposed surface and cross-section of the samples was performed using a stereo microscope Olympus SZ-CTV, Olympus, Tokyo, Japan and Quick PHOTO Camera 3.2 software and an inverted metallographic microscope Olympus GX51, Olympus, Tokyo, Japan with a motorized sample holding table using Stream Motion Version 2.3 software. A electron microscope was used to obtain Scanning Electron Images (SEIs) and to perform Energy Dispersive Spectroscopy (EDS) JEOL 6500 Thermal Field Emission SEM JSM-6500F, Inc., Peabody, MA, USA on the cross-section of the exposed samples (Figure 4b).

To evaluate the effect of degradation on the mechanical properties of the exposed samples, microhardness measurements were performed on the previously prepared cross-section in the degradation zone (Figure 4b). The microhardness tests were performed using a microhardness tester LEICA VMHT, Leica Microsystems, Bendheim, Germany according to the Vickers method. During indentation, a force of 0.980 N was applied for a duration of 15.0 s.

The degradation behavior and stability of the Al-2.18Mg-1.92Li alloy in a severe degradation condition was evaluated by measuring the difference in mass of the samples, the changes in pH and chemistry of the EXCO solution and changes in the microstructure and microhardness after 5, 24, 48 and 72 h of exposure. The degradation mechanism was defined by comparing the initial state of the alloy with the states of the samples after exposure.

## 3. Results

The chemical composition of the synthesized alloy and the Li/Mg ratio are shown in Table 2.

The hypoeutectic Li content (1.92 wt.%) suggests that the solidification will begin with the development of a primary α_Al_ dendritic network. The further progress of the solidification sequence will be determined by the low Li/Mg ratio (0.88) following Equation (2). The final microstructure of the synthesized Al-2.18Mg-1.92Li alloy will consist of an α_Al_ dendritic network and an Al_2_LiMg (T) phase with the solidification of transitional Al_2_Li (δ’) phase. Due to the complex behavior of Li and Mg during microstructure development, the retention of the transitional metastable Al_3_Li (δ’) phase is to be expected.

**Figure 5:**
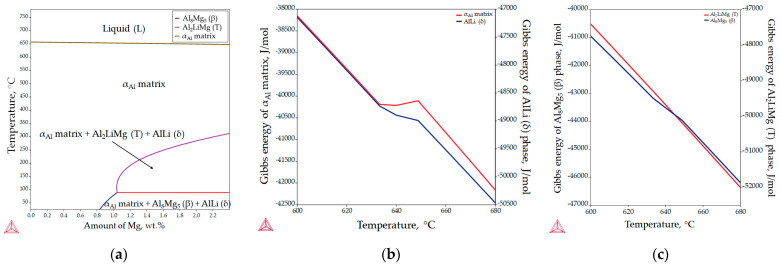
The equilibrium phase calculations for the Al-2.18Mg-1.92Li alloy: (**a**) the Al-rich corner of the equilibrium phase diagram, (**b**) Gibbs energy calculations for the α_Al_ matrix and AlLi (δ) phase and (**c**) Gibbs energy calculations for the Al_2_LiMg (T) and Al_8_Mg_5_ (β) phases.

The aluminum-rich corner of the equilibrium phase diagram for the Al-2.18Mg-1.92Li alloy is shown in Figure 5a, while the changes in Gibbs energy during solidification are illustrated in Figure 5b,c. The invariant reactions and the corresponding temperatures are listed in Table 3. The results of the equilibrium solidification sequence obtained with the Thermo Calc 2022a software support differ from the solidification sequence described in the literature [24]. According to the thermodynamic calculations, the equilibrium solidification sequence begins with the solidification of the primary α_Al_ dendritic network at 650.5 °C (Figure 5a, Table 3). The further microstructure development under equilibrium conditions is based on the solid-state reactions and the diffusion of the components (Al, Mg and Li) in the α_Al_ matrix (Figure 5a). The precipitation of the AlLi (δ) phase from the α_Al_ matrix begins at a temperature of 348.9 °C (Figure 5a, Table 2). The precipitation of Al_2_LiMg (T) phase at a temperature of 282.9 °C results from the reaction between the α_Al_ matrix and the previously precipitated AlLi (δ) phase (Figure 5a, Table 3). The microstructure development ends with the precipitation of the Al_8_Mg_5_ (β) phases at 81.9 °C (Figure 5a, Table 3). The reprecipitation and growth of the AlLi (δ) phase at the end of microstructure development is also indicated (Figure 5a, Table 3).

The spontaneous solidification and microstructure development in the Al-2.18Mg-1.92Li system is indicated by the negative values of the Gibbs energy for all phases in the temperature range from 600.0 to 680.0 °C. Although the initial Gibbs energy calculation was performed for the entire solidification range (from 780.0 °C to 25.0 °C), Thermo Calc 2022a allows the thermodynamic consideration of the Gibbs energy to an approximate minimum temperature of 500.0 °C. Moreover, in the temperature range from 633.0 to 648.0 °C, deviations in the Gibbs energy were observed for the α_Al_ matrix, and the AlLi (δ) and Al_8_Mg_5_ (β) phases. Considering that the equilibrium solidification results show the interaction between the α_Al_ matrix and the AlLi (δ) phase, deviations in the Gibbs energy were expected. However, as it is the last precipitating phase, this deviation was not expected for the Al_8_Mg_5_ (β) phase (Figure 5c). These results suggest a possible influence of the AlLi (δ) phase reprecipitation on the Al_8_Mg_5_ (β) phase development (Table 3). The more negative Gibbs energy values calculated for the AlLi (δ) and Al_2_LiMg (T) phases indicate their greater stability compared to the α_Al_ matrix and the Al_8_Mg_5_ (β) phase.

**Figure 6:**
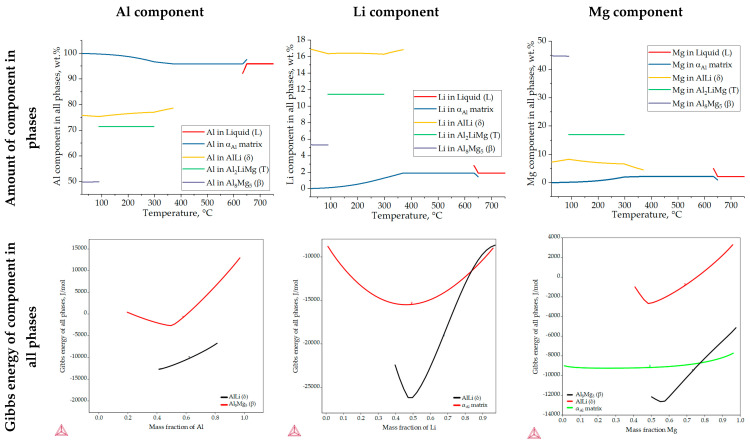
The results of the one axis equilibrium calculation of component distribution in all phases and the calculation of the Gibbs energy dependance on the mass fraction of the components at a temperature of 25.0 °C.

Figure 6 shows the results of the one axis equilibrium calculation of the component distribution in all the phases and the calculation of the Gibbs energy as a function of the mass fraction of the components at a temperature of 25.0 °C. The consideration of the component distribution in the liquid (L) phase stopped with the development of the α_Al_ dendritic network, confirming that it is the only solidifying phase in the Al-2.18Mg-1.92Li alloy system (Figure 6). The precipitation of intermetallic phases leads to a decrease in the content of Mg and Li in the α_Al_ matrix (Figure 6). The Al_2_LiMg (T) phase retains the same chemical composition of 71.5 wt.% Al, 11.4 wt.% Li and 17.1 wt.% Mg until the temperature reaches 89.1 °C when it stops precipitating (Figure 6). A similar behavior is exhibited by the Al_8_Mg_5_ (β) phase, which maintains a chemical composition of 27.8 wt.% Al, 44.7 wt.% Mg and 5.4 wt.% Li from 89.1 °C (Table 3) to the end of the solidification sequence (Figure 6). The influence of Mg on reducing the solid solubility of Li in the α_Al_ matrix as well as its tendency to substitute Li atoms in the binary Al-Li phase lattice can be observed at the end of the solidification sequence, when the precipitation of the Al_8_Mg_5_ (β) phase leads to an increase in the Li content in the AlLi (δ) phase from 16.3 wt.% to 17.0 wt.%, while the Mg content decreases from 8.3 wt.% to 7.2 wt.% (Figure 6). This redistribution of components can explain the deviation in the Gibbs energy of the Al_8_Mg_5_ (β) phase in the temperature range of 633.0 to 648.0 °C (Figure 5c). The one axis equilibrium calculation showed no bulking of the α_Al_ matrix with Li or Mg at the end of solidification (Figure 6).

The dependence of the Gibbs energy on the mass fraction of the components shows the stability of the α_Al_ matrix, and the AlLi (δ) the Al_8_Mg_5_ (β) phases. Spontaneous development and retention of the Al_2_LiMg (T) phase is not expected under the conditions defined in the calculation. The lowest Gibbs energy value of the α_Al_ matrix occurs at a composition of 48.0 wt.% Li, 42.0 wt.% Al and 10.0 wt.% Mg. Given that their Gibbs energy is negative for the entire composition range, this calculation suggests the bulking of the α_Al_ matrix with both components. The highest probability of AlLi (δ) phase formation is for the composition of 49.0 wt.% Li, 48.0 wt.% Mg and 51.0 wt.% Al. However, since its Gibbs energy is higher and trends towards positive values at higher compositions, the probability of Mg participating in the formation of the AlLi (δ) phase is minimal. A similar assumption applies to the participation of the Al component in Al_8_Mg_5_ (β) phase formation, where the lowest Gibbs energy value was obtained for 55.0 wt.% Mg and 45.0 wt.% Al. For higher amounts of Al, the Gibbs energy has a positive value. The chemical composition of the intermetallic phases calculated using the one axis equilibrium calculator does not match the composition with the lowest Gibbs energy, implying the possibility of phase decomposition at the temperature of the degradation testing (25.0 °C).

**Figure 7:**
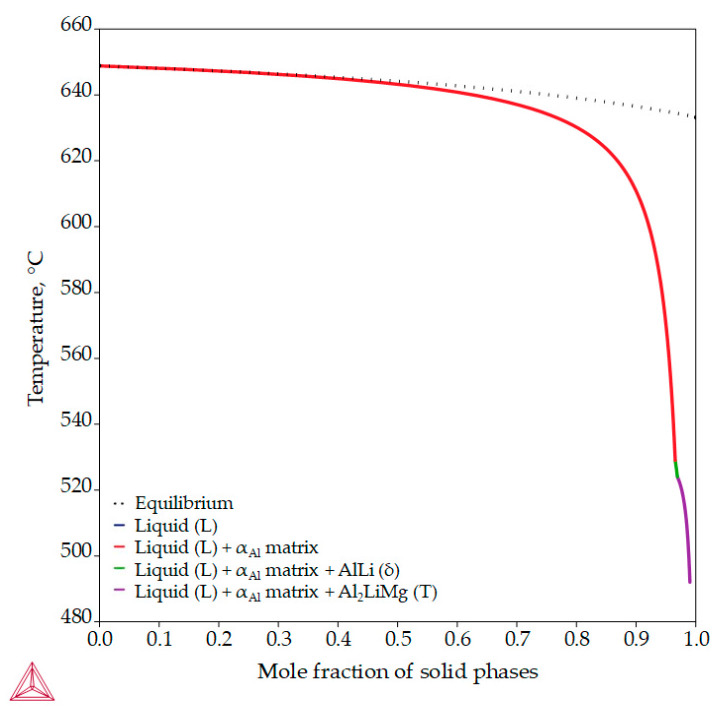
The non-equilibrium solidification sequence of an Al-2.18Mg-1.92Li alloy with a specified solidification range.

Unlike previous thermodynamic calculations, classical Scheil calculations stop with the complete solidification of the liquid (L) phase. In classic Scheil calculations, the chemical composition of the individual volume elements of the solid phase remains the same throughout the solidification process. Apart from the Al_8_Mg_5_ (β) phase, the non-equilibrium calculations assume the development of the same intermetallic phases (Figure 7, Table 4) as the equilibrium calculations but at higher temperatures (Figure 5). The non-equilibrium solidification sequence begins with the development of the primary α_Al_ dendritic network at 648.7 °C and the solidification of the AlLi (δ) phase at 548.7 °C and ends with the solidification of the Al_2_LiMg (T) phase at 524.0 °C (Table 4). In addition, the Al-2.18-1.92Li alloy exhibits a wide solidification range with a larger temperature gradient and degree of undercooling (Figure 7, equilibrium solidification line). The solidification under these circumstances enables microstructure refinement and improvements in alloy properties and microstructure degradation resistance.

**Figure 8:**
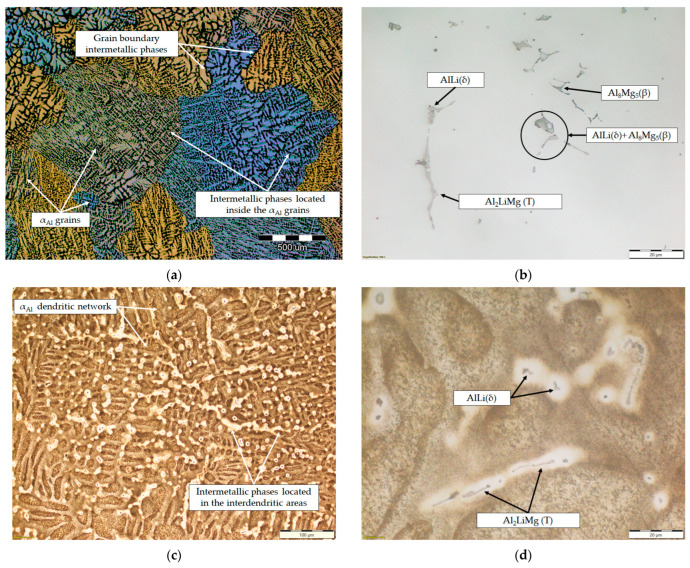
The results of light microscopy of the sample before degradation testing: (**a**) macrostructure of the sample, (**b**) microstructure of the sample in the polished condition, (**c**) microstructure of the sample after etching and (**d**) localization of intermetallic phases in the etched microstructure.

The microstructure of the samples before the degradation test are shown in Figure 8. The macrostructure of the sample consisted of equiaxed grains with heterogeneous grain sizes (Figure 8a). The macrostructure analysis also indicated the presence of intermetallic phases located within the α_Al_ grains and at the grain boundaries (Figure 8a). The intermetallic phases were evenly distributed with no PFZs near the grain boundaries (Figure 8a). The microstructural analysis of the as-polished samples allowed the identification of the intermetallic phases as an AlLi (δ) phase with a globular morphology, an Al_2_LiMg (T) phase with a rod-like morphology and an Al_8_Mg_5_ (β) phase with an irregular morphology and dark in color (Figure 8b). In the microstructure of the polished sample, a combined AlLi (δ) + Al_8_Mg_5_ (β) phase was observed, pointing to their parallel formation at the end of the solidification sequence. This transition phase formation confirms the results of the one axis equilibrium calculation of the Mg and Li redistribution between the AlLi (δ) and Al_8_Mg_5_ (β) phases (Figure 6) and the deviation in the Gibbs energy in the same temperature range (Figure 5b,c). After etching, a primary α_Al_ dendritic network with well-developed branches was observed in the microstructure of the sample (Figure 8c). The intermetallic phases identified in the polished condition were distributed in the interdendritic areas (Figure 8d).

The effects of the exposure time on the samples’ physical properties and calculated degradation rate are shown in Table 5. During degradation under severe environmental conditions, a decrease in the mass of the samples was observed. The mass losses (∆m) increased with increasing exposure time, up to a period of 72 h. The highest degradation rate of 9.3·10^−5^ g/s was calculated for an exposure time of 5 h (Table 5). Prolongation of the exposure time resulted in a reduction in the degradation rate. Considering that the calculations are based on mass loss, the decrease in degradation rate may be a result of the formation and retention of degradation products on the exposed surface. The changes in the pH and chemistry of the EXCO solution as a function of the exposure time are shown in Table 6.

**Figure 9:**
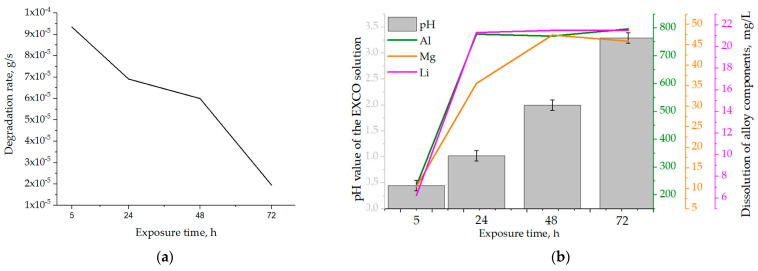
The influence of exposure time on the (**a**) degradation rate and (**b**) solution’s pH and dealloying.

Although the pH of the EXCO solution remained acidic throughout the test period, it increased with increasing exposure time (Table 6) due to ion exchange between the exposed surface of the samples and the environment. Based on the literature [38], pH values outside the range of 3 to 10 will lead to cavity formation on the exposed surface, allowing further microstructure degradation. From the dependence of the degradation rate on the exposure time shown in Figure 9a, it can be concluded that the degradation rate decreased with the increase in exposure time. The obtained results are in agreement with the data available in the literature [43], identifying the pH of 0.4 as critical for Al-Li and Al-Li-X (where X is an additional alloying element) alloys.

The increase in the amount of Al, Mg and Li in the EXCO solution after exposure of the samples shows the occurrence of rapid anodic and cathodic reactions, which lead to degradation of the microstructure and dealloying (Table 6). For all exposure times, the dissolution of Al was the highest, followed by the dissolution of Mg and Li (Figure 9b, Table 6).

**Figure 10:**
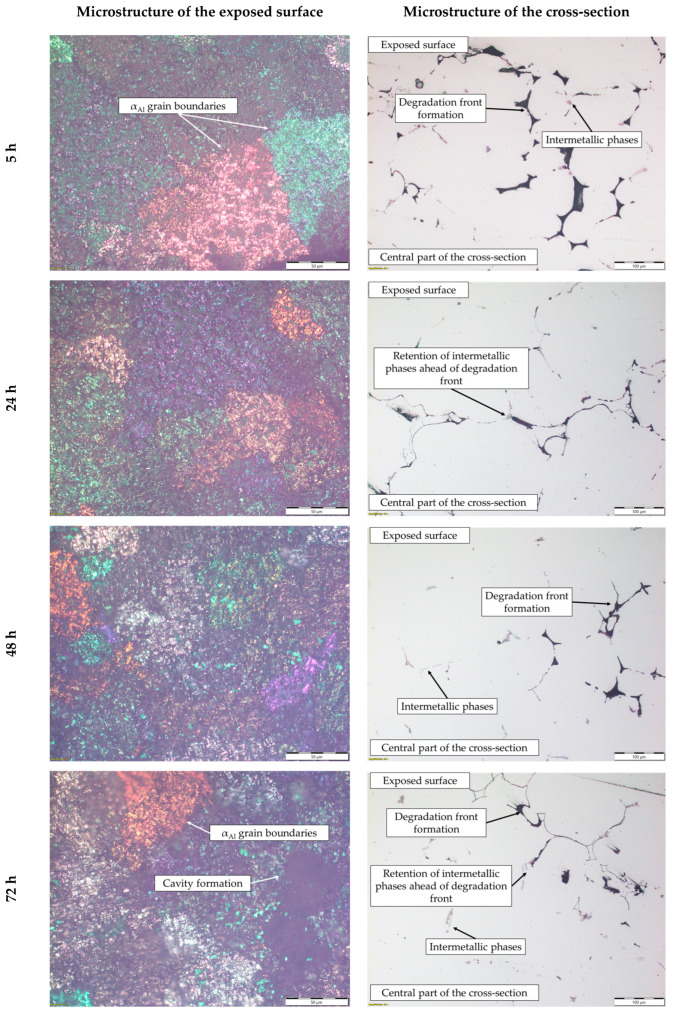
The microstructure of the exposed surface and cross-section of the sample after degradation testing.

**Figure 11:**
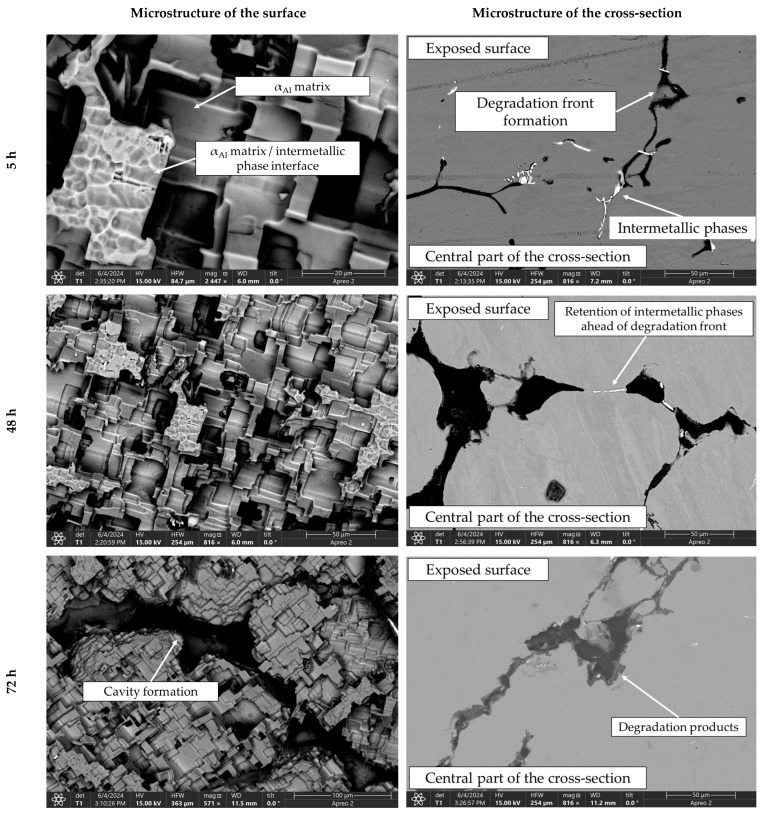
SEIs of the exposed surface and cross-section of the sample after 5, 48 and 72 h of degradation.

These results indicate that dealloying and microstructural degradation are most likely associated with the dissolution of the α_Al_ matrix. The impact of the degradation time on the microstructure is shown in Figure 10 and Figure 11.

The dissolution of Al and Li was highest in the first 24 h and at lower pH values (Figure 9b, Table 6). During the exposure period of 24 to 72 h, the loss of Al and Li components was almost constant. In contrast, the Mg content in the EXCO solution increased up to an exposure time of 48 h (Figure 9b, Table 6). This behavior of the components suggests that microstructural degradation and dealloying most likely occur through an anodic dissolution mechanism. The increase in the Mg and Li contents in the EXCO solution may be a result of the bulked α_Al_ matrix dealloying, dissolution of the intermetallic phases or the detachment of the intermetallic phases from the matrix due to the instability and wedge effect of the degradation products. Considering the results of the one axis equilibrium calculations of the component distribution as well as the equilibrium calculation of the Gibbs energy, the increase in the Mg and Li contents due to the dissolution of the bulked α_Al_ matrix is unlikely. On the other hand, the higher amount and the continuous increase in Mg over a longer exposure time (up to 48 h) indicate a higher stability of the Al_8_Mg_5_ (β) phases.

**Figure 12:**
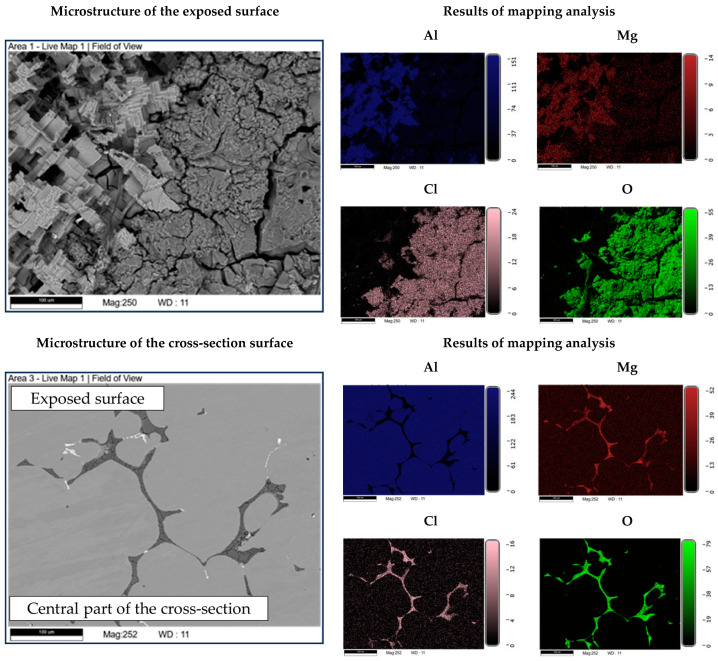
SEIs of the exposed surface and cross-section of the sample with corresponding mapping analysis.

The microstructure of the exposed surface shown in Figure 10 reveals a uniform degradation within the α_Al_ grains and along the grain boundaries. The results of the scanning electron microscopy of the exposed surface confirmed uniform dissolution of the α_Al_ matrix with the local appearance of dimples caused by the dislodgement of the intermetallic phases (Figure 11, microstructure of the samples’ surface). The appearance of a stronger localized degradation in the form of cavities was noticeable after 72 h of degradation (Figure 11). The mapping analysis at higher magnification revealed that the decomposition products of the surface consisted of chlorine and oxygen, with an approximate chemical composition of 54.0 wt.% Al, 36.3 wt.% O, 7.8 wt.% Cl and 1.5 wt.% Mg (Figure 12, microstructure of the surface).

The microstructural analysis performed on the cross-section surface showed that the degradation started at the interface between the α_Al_ matrix and the intermetallic phase (Figure 11, microstructure of the cross-section). Extending the exposure time led to a progression of degradation along the interface and the formation of cavities (Figure 11, microstructure of the cross-section). Since the intermetallic phases were preserved ahead of the degradation front, it can be assumed that the microstructural degradation was caused by an anodic mechanism, followed by a detachment of the intermetallic phases due to the instability of the degradation products (Figure 11). The results of the quantitative analysis identified the degradation products as 90.4 wt.% Al, 5.3 wt.% O, 3.1 wt.% Mg and 0.7 wt.% Cl (Figure 12, microstructure of the cross-section).

Table 7 presents the microhardness values obtained from the cross-section of the samples, both prior to and following the degradation tests.

By maintaining the microhardness value throughout the whole exposure period, the Al-2.18Mg-1.92Li alloy showed a high stability in the degradation area (Table 7). An increase in the microhardness values after exposure indicates an increase in the stress around the degradation front due to the wedge effect of the degradation products. The high values of the standard deviation, calculated for the 5, 48 and 72 h exposure time, indicates a local instability of the α_Al_ matrix in the vicinity of the degradation products (Table 7).

## 4. Discussion

The review of the literature indicated that the chemical composition of the environment will dictate the mechanism of Al-Mg-Li alloy degradation. Depending on the pH value, microstructural degradation will most likely begin on the surface of the alloy with the formation of cavities [30]. These cavities will serve as an initiator for the development of other degradation processes. Based on the metallurgical conditions and microstructure of the alloy, the next phase of the degradation may occur intergranularly or exfoliatively [38]. While intergranular degradation will transpire either according to the anodic dissolution model or the PFZ breakdown model, the condition for the onset and progression of exfoliation degradation is the existence of a highly directional grain structure [41]. Given that the degradation testing was performed on the samples in the as-cast condition, the exfoliation degradation mechanism is highly unlikely [30]. However, previous investigations of the solidification sequence and microstructure development of Al-Mg-Li alloy systems have indicated the formation of anodic intermetallic phases as well as the development of PFZs [34,44,45].

A previous study of Al-2.18Mg-1.92Li alloy degradation behavior was performed electrochemically using the time dependance of the open-circuit potential and Tafel polarization curves. The use of electrochemical measurements allowed for the comparison of the stability, degradation potential and degradation rate of the samples in as-cast and solutionized conditions. The measurement results indicated a less negative corrosion potential (−749.84 mV), lower current density (1.52 10^3^ µA cm^2^), anodic slope (38.60 mV/dec), cathodic slope (296.26 mV/dec) and degradation rate (17.01 mm/year) for the as-cast sample. Nevertheless, microstructural degradation was observed. Light microscopy performed on the surface and on cross-section of the samples after degradation indicated surface cavity formation, followed by intercrystalline degradation involving Al_8_Mg_5_ (β), Al_2_LiMg (T) and the α_Al_ matrix. However, the use of single short-term electrochemical measurement method did not enable the determination of the exact degradation and dealloying mechanism [43].

Therefore, an additional experiment was designed to achieve sequential long-term tracking of alloy behavior under severe environmental conditions. The simulation of different types of outdoor exposures, according to the ASTM G34-01 standard [42], involved continuous immersion of the test samples in a solution with a defined chemical composition. The long-term exposure of the Al-2.18Mg-1.92Li alloy to the EXCO solution enabled the sequential measurement of the mass of the samples and the chemical composition of the solution after 5, 24, 48 and 72 h. The correlation between the obtained results indicated the degradation susceptibility. The effect of the microstructure first on the acceleration of the degradation process and then on the passivation was estimated by combining the results of Thermo Calc’s 2022a thermodynamic calculations with the results of the various metallographic analysis techniques. The degradation mechanism was identified by comparing the microstructure of the sample before and after degradation.

Based on the chemical composition, a solidification sequence characteristic for an alloy with hypoeutectic Li was assumed. This microstructure development involved the development of a primary α_Al_ dendritic network and peritectic solidification of the Al_2_LiMg (T) phase involving the prior solidification of a metastable Al_3_Li (δ’) phase. The thermodynamic considerations of the equilibrium and non-equilibrium solidification sequence confirmed the development of anodic intermetallic phases and extended the microstructure development to include an Al_8_Mg_5_ (β) phase. Calculations of the component distribution within the intermetallic phases indicated a low probability of α_Al_ bulking and PFZ development. The dependance of the Gibbs energy on temperature indicated component redistribution between the intermetallic phases during solidification and a higher stability of the intermetallic phases compared to the matrix. The higher stability of the intermetallic phases was also confirmed by the dependance of the Gibbs energy on the mass fraction of the component. However, it was determined that chemical composition of the intermetallic phases calculated by the one axis equilibrium calculator does not correspond to the most stable phase composition. Although the results of the thermodynamic calculations indicated a higher probability of degradation occurring through the anodic dissolution mechanism due to the lower stability of the intermetallic phases, it is questionable whether their dissolution will occur during degradation. This assumption was further confirmed by the results of the metallographic analysis of the initial sample structure. The initial macrostructure of the sample consisted of equiaxed grains with heterogeneous grain sizes. The macrostructure analysis also indicated the presence of intermetallic phases located within the α_Al_ grains and at the grain boundaries with no PFZs present. The microstructural analysis of the samples allowed for the identification of the intermetallic phases as AlLi (δ), Al_2_LiMg (T) and Al_8_Mg_5_ (β), which were located in the interdendritic areas of the α_Al_.

**Figure 13:**
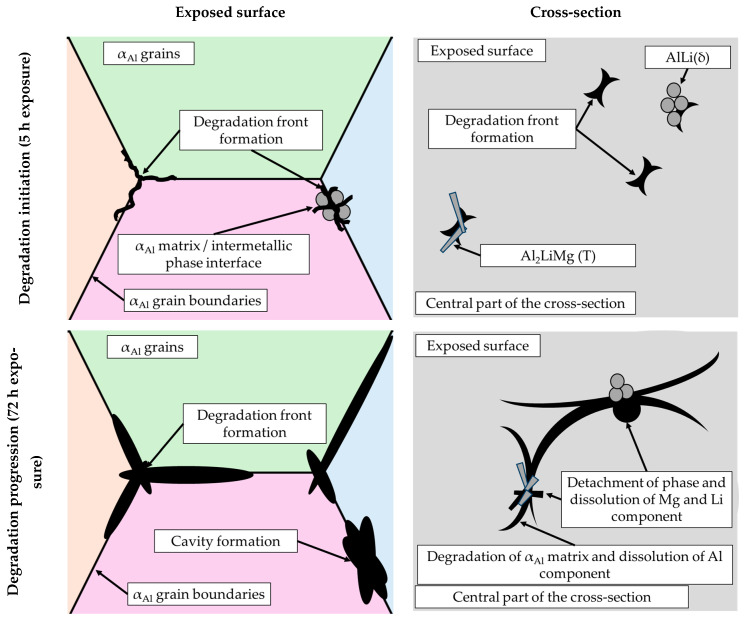
A schematic representation of the degradation mechanism of the Al-2.18Mg-1.92Li alloy.

By considering the influence of degradation time on the physical properties of the samples, an increase in mass loss with increasing degradation time was observed. The increase in the pH value of the solution from an initial 0.4 to 3.3 was also observed. The highest degradation rate of 9.3 10^−5^ g/s was calculated for a degradation time of 5 h. A further increase in the exposure time resulted in a decrease in the degradation rate. These results are in accordance with the data available in the literature indicating that Al-Li alloys are most sensitive to a pH of 0.4 [38]. The results of the ICP-MS analysis of the solutions after degradation indicated the occurrence of dealloying and dissolution of all three components. Aluminum experiencing the highest dissolution of all the components indicates that the microstructure degradation and dealloying most likely occur through the dissolution of the α_Al_ matrix, while the increase in the other two components may be a consequence of intermetallic phase detachment or dissolution of the bulked α_Al_ matrix. Considering the conclusions of previously used methods, the first mechanism is the most likely [42]. A schematic representation of the degradation mechanism of the Al-2.18Mg-1.92Li alloy is shown in Figure 13. This was confirmed by the results of the light microscopy and scanning electron microscopy performed on the exposed surface as well as on the cross-section of the samples after exposure. The microstructure of the exposed surface revealed uniform degradation within the α_Al_ grains and along the grain boundaries without visible cavity formation (Figure 13, microstructure of the exposed surface). The results of the scanning electron microscopy of the exposed surface confirmed uniform dissolution of the α_Al_ matrix with the local appearance of dimples caused by the detachment of the intermetallic phases (Figure 11 microstructure of the exposed surface). The appearance of a stronger localized degradation in the form of cavities was noticeable after 72 h of degradation (Figure 11, microstructure of the exposed surface, 72 h exposure). Although the formation of the cavities was expected much earlier, this observation is not consistent with the data available in the literature that indicates the formation of cavities outside the pH range of 3 to 10 [38]. The microstructural analysis of the cross-section surface showed that the degradation started at the interface between the α_Al_ matrix and the intermetallic phases (Figure 13, microstructure of the cross-section, degradation initiation). Extending the exposure time led to the degradation progression along the interface and the formation of cavities (Figure 13, microstructure of the cross-section, degradation progression). Since the intermetallic phases were preserved ahead of the degradation front, it can be assumed that the microstructural degradation was caused by an anodic mechanism, followed by a detachment of the intermetallic phases due to the instability of the degradation products (Figure 13, microstructure of the cross-section, degradation progression). The values of the standard deviation for the microhardness measured after 5, 48 and 72 h of exposure time showed and confirmed the local instability of the α_Al_ matrix near the degradation products, while the increase in the microhardness value of the samples after degradation indicated an increase in stress and the wedging effect of the degradation products. The metallographic analysis of the samples after the electrochemical corrosion test and the Tafel extrapolation method showed that the degradation started with the formation of cavities on the surface of the sample and progressed intergranularly, involving both intermetallic phases and the α_Al_ matrix [44]. Compared to previously performed electrochemical tests, the results obtained by immersing the samples in the EXCO solution allowed for the identification of the degradation mechanism as an anodic. Microstructure analysis revealed that the intergranular degradation started at the interface between the α_Al_ matrix and the intermetallic AlLi (δ), Al_2_LiMg (T) and Al_8_Mg_5_ (β) phases. The degradation progression and the detachment of the intermetallic phases are the result of a local instability induced by the wedge effect of the degradation products.

Characterization of the physical properties and microstructure of the Al-2.18Mg-1.92Li alloy samples before and after degradation testing partially identified the anodic mechanism as the basis of the microstructure degradation. In order to assess the degradation susceptibility of the alloy in more detail, it is necessary to repeat the tests after the complete removal of the degradation products in an ultrasonic bath. In this way, more accurate data on the degradation rate can be obtained and the influence of the degradation products on the stability of the alloy can be considered. It is also necessary to redesign the experimental setup to take into account the more specific pH value intervals that have been identified as critical for Al-Li alloys. This can be achieved by neutralizing the EXCO solution and setting a higher initial pH.

## 5. Conclusions

This investigation evaluated the degradation behavior of Al-2.18Mg-1.92Li alloy under the severe environmental conditions defined by ASTM G34-01 standard. The alloy’s tendency towards degradation was assessed by characterizing the physical properties of the samples and measuring the changes in the solution’s pH and chemistry. The degradation mechanism was determined by comparing the results of the initial microstructure with the microstructure of the samples after 5, 24, 48 and 72 h of exposure. The impact of the severe environmental conditions on the mechanical properties was estimated by measuring the microhardness of the samples before and after degradation.

The results of the equilibrium and non-equilibrium solidification sequence indicated the development of a primary α_Al_ dendritic network, followed by solidification of the Al_2_LiMg (T), AlLi (δ) phase and Al_8_Mg_5_ (β) phase. Calculations of component distribution within the intermetallic phases indicated a low probability of α_Al_ bulking and PFZ development. The dependance of the Gibbs energy on temperature indicated a higher stability of the intermetallic phases compared with the α_Al_ matrix. While the macrostructure of the sample before exposure indicted equiaxed grains with heterogeneous grain sizes, the microstructure analysis indicated the presence of intermetallic phases located within the α_Al_ grains and at the grain boundaries with no PFZs present. The intermetallic phases were identified as AlLi (δ), Al_2_LiMg (T) and Al_8_Mg_5_ (β), which were located in the interdendritic areas and grain boundaries.

By correlating the mass loss with the exposure time, the highest degradation rate of 9.3 10^−5^ g/s was calculated for an exposure time of 5 h and a solution pH of 0.4. The minimum degradation rate of 1.9 10^−5^ g/s was calculated for an exposure time of 72 h at a measured pH value of 3.29.

The changes in the chemical composition of the EXCO solution indicated the occurrence of dealloying and microstructure degradation involving the α_Al_ matrix and intermetallic phases. The microstructural characterization of the exposed surface and the cross-section of the samples indicated the occurrence of intergranular degradation during the exposure. The identification of the α_Al_/intermetallic phase interface as the degradation initiation site as well as the retention of the intermetallic phases ahead of the degradation front pointed to an anodic dissolution mechanism for the intergranular degradation.

In order to assess the degradation susceptibility of the alloy in more detail, it is necessary to redesign the experimental setup to take into account the more specific pH value intervals that have been identified as critical for Al-Li alloys, and subject the alloy to heat treatment by solutionizing, precipitation hardening or artificial aging. In this way, it will be possible to assess the influence of individual microstructural constituents on the degradation stability of the alloy.

## Figures and Tables

**Table 1:** The parameters used to evaluate the degradation susceptibility of the Al-2.18Mg-1.92Li alloy during EXCO testing.

Parameter	Unit	Description
m_S_	g	initial mass of the sample
m_E_	g	mass of the sample after degradation
∆m	g	difference in mass resulting from the degradation of the sample
t_EXP_	s	degradation exposure time
pH_S_	/	initial pH value of EXCO solution
pH_E_	/	pH value of the EXCO solution after the degradation
v_cor_	g/s	degradation rate
/	/	change in the chemical composition of the EXCO solution after degradation

**Table 2:** The chemical composition of the synthesized alloy.

Chemical Composition, wt.%			
Li	Mg	Al	Li/Mg Ratio
1.92 ± 0.06	2.18 ± 0.07	balance	0.88

**Table 3:** The invariant reactions calculated using one axis equilibrium calculation.

Reaction Number	Reaction	Temperature, °C	Amount of Component, wt.%		
			Al	Mg	Li
1.	$\mathrm{Liquid}\;(L)\;\to {\;\alpha }_{\mathrm{Al}}$	650.5	0.0838	0.0011	0.0007
2.	$\alpha_{\mathrm{Al}}\to \;\mathrm{AlLi}(\delta )$	348.9	0.0041	0.0008	0.0002
3.	$\alpha_{\mathrm{Al}}+\mathrm{AlLi}\left(\delta \right)\to {\mathrm{Al}}_{2}\mathrm{LiMg}(T)$	282.9	0.0022	0.0003	0.0005
4.	$\alpha_{\mathrm{Al}}\to {\mathrm{Al}}_{8}{\mathrm{Mg}}_{5}(\beta )+\mathrm{AlLi}\left(\delta \right)$	89.1	0.0119	0.0012	0.0107

**Table 4:** The invariant reactions calculated for non-equilibrium solidification.

Reaction Number	Reaction	Temperature, °C
1.	$\mathrm{Liquid}\;(L)\;+\;\alpha_{\mathrm{Al}}$	648.7
2.	$\mathrm{Liquid}\;(L)\;+\;\alpha_{\mathrm{Al}}+\mathrm{AlLi}(\delta )$	548.7
3.	$\mathrm{Liquid}\;(L)\;+\;\alpha_{\mathrm{Al}}+{\mathrm{Al}}_{2}\mathrm{LiMg}(T)$	524.0

**Table 5:** Dependence of the physical properties of the tested samples on the exposure time.

T_EXP_, h	M_S_, g	M_E_, g	∆m, g	v_cor_, 10^−5^ g/s
5	12.1380	12.1312	0.06	9.3
24	11.1727	11.1505	0.20	6.9
48	8.7996	8.7693	0.35	5.9
72	13.6626	13.6396	0.17	1.9

**Table 6:** Influence of the exposure time on the properties of the EXCO solution.

t_EXP_, h	pH_S_	pH_E_	Chemical Composition, mg/L		
			Al	Mg	Li
5	0.4	0.5	233 ± 6.99	10.4 ± 0.31	6.24 ± 0.18
24	0.4	1.0	778 ± 23.4	35.6 ± 1.06	21.3 ± 0.64
48	0.4	2.0	771 ± 23.1	47.4 ± 1.42	21.5 ± 3.21
72	0.4	3.3	797 ± 23.9	45.9 ± 1.38	21.5 ± 3.21

**Table 7:** Microhardness measured near the exposed surface of the samples after degradation.

Exposure Time, h	Number of Measurements	Mean Value, HV	Standard Deviation
Sample before degradation testing	3	63.1	1.4
5	5	73.1	5.5
24	3	75.7	1.7
48	5	72.7	3.2
72	5	75.8	6.7

## Data Availability

The original contributions presented in this study are included in the article. Further inquiries can be directed to the corresponding authors.
